# Determining the Number of Graphene Nanoribbons in Dual-Gate Field-Effect Transistors

**DOI:** 10.1021/acs.nanolett.3c01931

**Published:** 2023-09-06

**Authors:** Jian Zhang, Gabriela Borin Barin, Roman Furrer, Cheng-Zhuo Du, Xiao-Ye Wang, Klaus Müllen, Pascal Ruffieux, Roman Fasel, Michel Calame, Mickael L. Perrin

**Affiliations:** † Transport at Nanoscale Interfaces Laboratory, Empa Swiss Federal Laboratories for Materials Science and Technology, 8600 Dübendorf, Switzerland; ‡ nanotech@surfaces Laboratory, Empa Swiss Federal Laboratories for Materials Science and Technology, 8600 Dübendorf, Switzerland; § State Key Laboratory of Elemento-Organic Chemistry College of Chemistry, Nankai University, 300071 Tianjin, China; ∥ Max Planck Institute for Polymer Research, 55128 Mainz, Germany; ⊥ Department of Chemistry Biochemistry and Pharmaceutical Sciences, University of Bern, 3012 Bern, Switzerland; # Department of Physics, University of Basel, 4056 Basel, Switzerland; ∇ Swiss Nanoscience Institute, University of Basel, 4056 Basel, Switzerland; ◆ Department of Information Technology and Electrical Engineering, ETH Zurich, 8092 Zurich, Switzerland; ¶ Quantum Center, ETH Zürich, 8093 Zürich, Switzerland

**Keywords:** graphene, nanoribbons, field-effect transistors, charge-transport

## Abstract

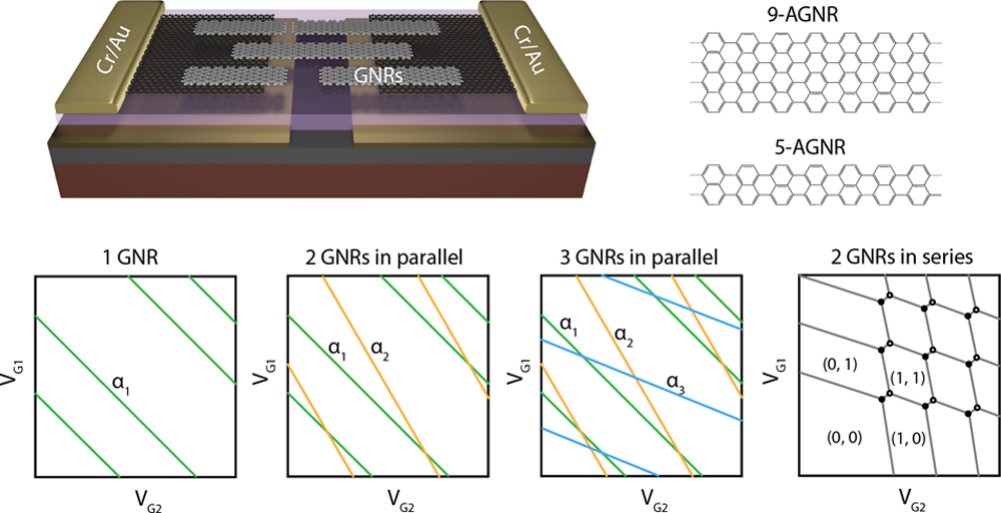

Bottom-up synthesized graphene nanoribbons (GNRs) are increasingly attracting interest due to their atomically controlled structure and customizable physical properties. In recent years, a range of GNR-based field-effect transistors (FETs) has been fabricated, with several demonstrating quantum-dot (QD) behavior at cryogenic temperatures. However, understanding the relationship between the cryogenic charge-transport characteristics and the number of the GNRs in the device is challenging, as the length and location of the GNRs in the junction are not precisely controlled. Here, we present a methodology based on a dual-gate FET that allows us to identify different scenarios, such as single GNRs, double or multiple GNRs in parallel, and a single GNR interacting with charge traps. Our dual-gate FET architecture therefore offers a quantitative approach for comprehending charge transport in atomically precise GNRs.

Quantum technologies utilize the quantum properties of matter to create innovative tools and devices, which require precise control of matter at the nanoscale for tailoring specific material properties. Developing material platforms with atomic control over their chemical structure is therefore essential for advancements in technologies such as quantum information processing, sensing, communication, and cryptography. Among them, atomically precise graphene nanoribbons (GNR) synthesized by bottom-up approaches are of particular interest. This is due to the accurate control over their physical properties at the atomic scale^1−3^ which endows them with a wide range of electronic,^4,5^ magnetic,^2^ and optical properties.^6^ In recent years, bottom-up synthesized GNRs have been integrated into several devices with the observation of Coulomb blockade,^7−9^ excited states,^7,9,10^ and Franck–Condon blockade.^9,10^ Quantifying the number and geometry of GNRs in a device channel is vital for technological applications, yet it remains a challenging task to accomplish by using a single-gate architecture, especially when multiple GNRs are contacted.

In this work, we contact single 5- and 9-atom-wide GNRs (5-AGNRs and 9-AGNRs) using graphene electrodes embedded within a dual gate architecture. We observe well-defined quantum transport behaviors, including Coulomb blockade and excited states, which reflect the intrinsic physical properties of the two types of GNRs being investigated. The presence of the two gates allows us to build a qualitative relationship between charge-transport features and the geometry of the GNRs in the device channel. In addition, we demonstrate that when a single QD is formed in a 9-AGNR, a Coulomb blockade persists up to temperatures as high as 250 K.

Figure 1a displays a schematic illustration of our armchair GNR device with a pair of graphene electrodes separated by a nanogap (15–25 nm). Two metal gates (G1 and G2) with a separation of 20 nm are defined under the source and drain graphene electrodes. The gates and electrodes are isolated from each other by a 20 nm Al_2_O_3_ dielectric layer. As the electronic coupling between the GNRs and the graphene is weak, we anticipate the formation of QDs at low temperatures,^7−11^ as illustrated in Figure 1b with an energy diagram of the graphene–GNR–graphene junction. The gates (G1 or G2), located at different lateral positions below the nanojunction, are employed to introduce asymmetric electrostatic gate fields in the junction. A scanning electron micrograph (SEM) of the as-fabricated device before GNR transfer is presented in Figure 1c, alongside a close-up of the nanogap region.

**Figure 1:**
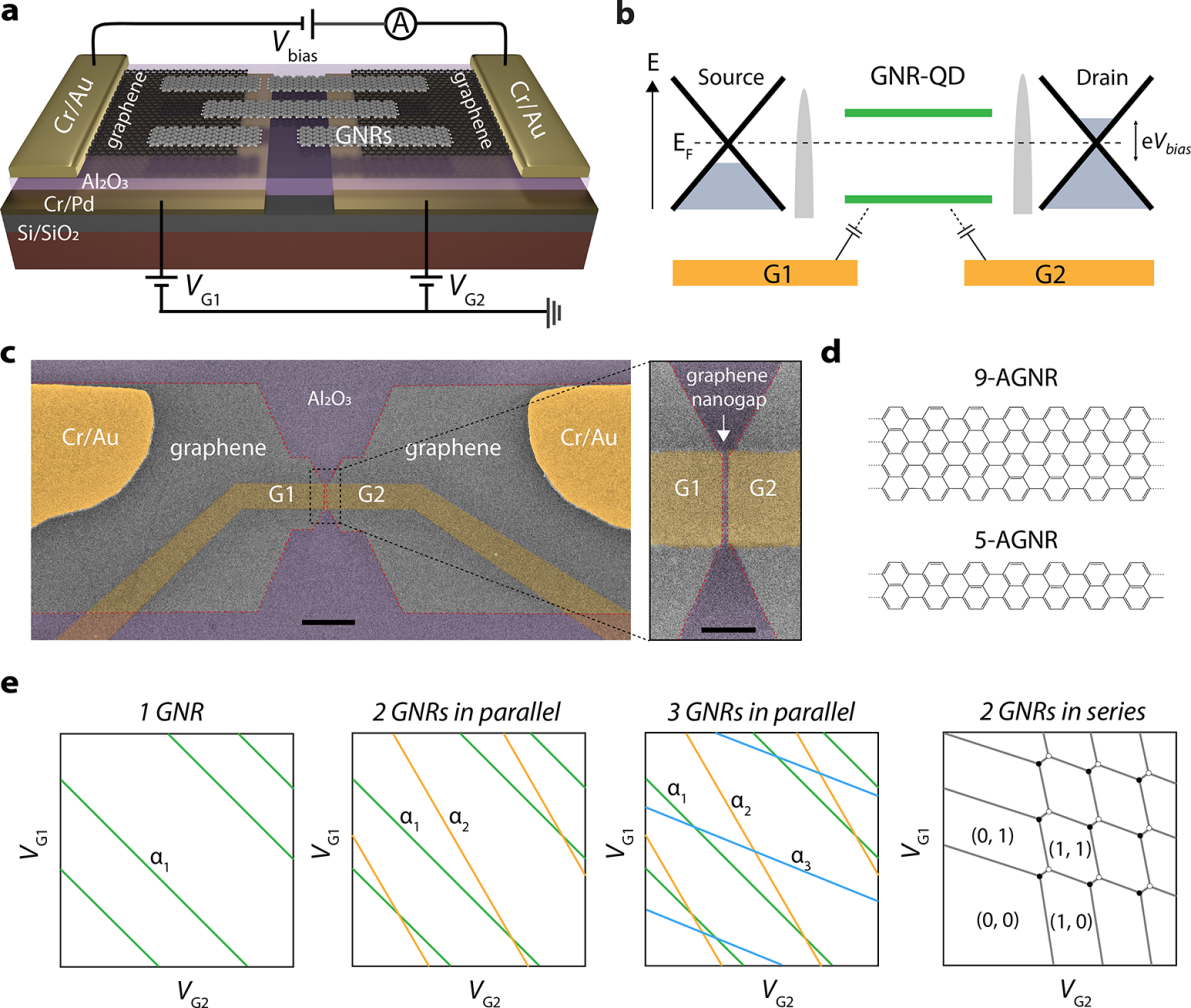
Dual-gate GNR quantum dot device. (a) Schematic illustration of the device architecture, including the measurement circuit. (b) Illustration of the band structure of GNR QD contacted by graphene electrodes, and two gates (G1 and G2) coupled to the QD differently in spatial. (c) Left: False colored scanning electron micrograph image of a device prior to GNR transfer, showing graphene electrode separated by ∼25 nm, dual gates G1 and G2 (10 nm thick) separated by ∼20 nm, and a 20 nm Al_2_O_3_ (purple) as dielectric. Scale bar: 1 μm. Right: close-up of the graphene nanogap region showing a good alignment between graphene gap and gate gap. Scale bar, 200 nm. (d) Structure of a 9-AGNR and a 5-AGNR backbone, showing their atomically precise width and edge structure. (e) Schematics of dual-gate current maps showing various scenarios, including one GNR, two GNRs in parallel, three GNRs in parallel, and two GNRs in series (from left to right). The colored lines within the map illustrate the resonances of GNRs.

In this work, two types of armchair GNRs are transferred on top of the graphene electrodes. The first type of GNR is the uniaxially aligned 9-AGNRs (Figure 1d, top), with lengths between 20 and 80 nm.^12^ The second type of GNR is the nonaligned 5-AGNRs (Figure 1d, bottom), with lengths between 5 and 25 nm.^13^ More details about the growth of the GNRs and device fabrication can be found in the Materials and Methods (Supporting Information).

For technological applications involving atomically precise GNRs, the development of a quantitative method for determining the number of GNRs in a junction is crucial. To address this, we developed a dual-gate FET architecture that enables the measurement of a dual-gate current map (current for fixed low bias as a function of *V*_G1_ versus *V*_G2_). Such a map is particularly suited for identifying the coupling of the GNR-based QDs to both gates. Under the assumption that no two GNRs possess the exact same coupling to both gates, we analyze the resonances in the dual-gate conductance maps and quantitatively determine both the number of the GNRs and their geometry (parallel or series). For instance, in a single QD system (Figure 1e, left), parallel resonances with the same slope will be observed, while for two or more noncoupled QDs in parallel, two (Figure 1e, middle left) or more (Figure 1e, middle right) sets of parallel running resonances will be discernible. For two GNRs in series (Figure 1e, right, not observed in this work) we anticipate the observation of the typical double QD features, such as the honeycomb structure in the dual-gate conductance maps at low bias and the bias triangles at high bias voltage.^14^

In the following, we will show that by combining the two measurement types several scenarios can be identified. In total, 9 devices are presented, 5 with 9-AGNRs (Devices 1, 2, 6, 7 and 9) and 4 with 5-AGNRs (Devices 3, 4, 5 and 8), all characterized at cryogenic temperatures of 4 or 10 K.

Figure 2a (middle panel) presents the stability diagram recorded on Device 1 on which uniaxially aligned 9-AGNRs have been transferred. In the negative gate range from −8 to 0 V (marked by a white dashed box), we observed several regular and closing Coulomb diamonds with addition energies *E*_add_ between 80 and 110 meV. At around *V*_G1_= 0 V, a large diamond with *E*_add_ of ∼380 meV is observed, which we attribute to the band gap of the 9-AGNR. As the electrodes are micron-sized, it is unlikely that the large diamonds stem from the graphene electrodes, in which current suppression can be observed at the Dirac point but typically when their size is sub-100 nm.^15^ Based on the position of the band gap, of which we estimate the size to be ∼380 meV, the number of holes in the dot is assigned. A few additional resonances with energies around 40 meV (green dashed line) are observed. Importantly, they run parallel to the diamond edges, pointing toward electronic or vibrational excited states originating from the 9-AGNR itself. This contrasts with states originating from the leads that result in resonances that do not run parallel to the diamond edges.^16^ Such lead states are not visible in our data. Interestingly, in the 1 h state, a nonzero conductance is present in the Coulomb blockade region. We attribute this to a signature of cotunneling, in line with the intermediate dot-lead coupling. Nevertheless, the possibility of this conductance arising from parasitic tunneling pathways cannot be ruled out.^16^

**Figure 2:**
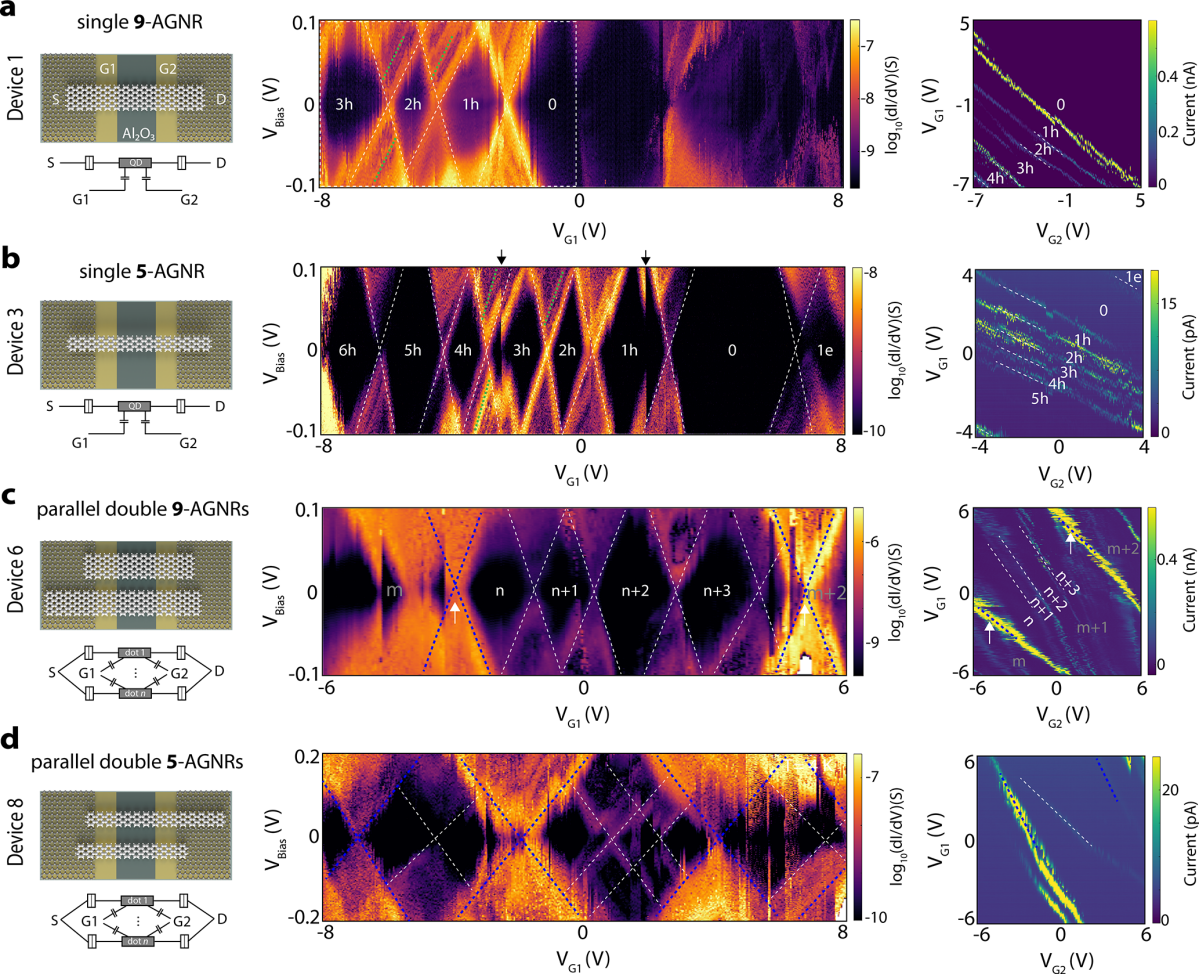
Determining the number of GNRs. (a, b) Transport measurements on single QD devices by contacting a single 9-AGNR (a) and a single 5-AGNR (b). Left: Illustrations of single QD geometries with their respective equivalent circuits. Middle: Stability diagrams were recorded at *V*_G2_ = 0 V and a temperature of 10 K (9-AGNR device) or 4 K (5-AGNR device). The number of electrons/holes in the QD is assigned. Right: low-bias (*V*_Bias_ = 0.6 mV) current as a function of *V*_G1_ and *V*_G2_, including several Coulomb resonances in parallel. (c, d) Transport measurements on two parallel-double QD devices with contact to two 9-AGNRs (c) and two 5-AGNR (d). Left: Illustration of two parallel QDs with their respective equivalent circuits. Middle: Stability diagrams recorded at *V*_G2_ = 0 V and a temperature of 10 K (9-AGNR device) or 4 K (5-AGNR device). The edges of diamonds originating from two different QDs are highlighted by blue and white dash lines. In panel c, a few Coulomb diamonds with sequential tunneling in one of the QDs are numbered with *n*, *n* + 1, *n* + 2, and *n* + 3. Right: Low-bias current (*V*_Bias_ = 1 mV) as a function of *V*_G1_ and *V*_G2_, including several Coulomb resonances in predominantly two slopes.

In the right panel of Figure 2a, we measured a dual-gate current map (*V*_Bias_ = 0.6 mV) for Device 1. Here, multiple diagonal resonances are observed, all with comparable slopes. These resonances correspond to the Coulomb peaks in the hole-transport regime of the QD. From the slope, we extract the relative gate coupling α_G1_/α_G2_ = 1.2. From the stability diagram and dual-gate current map, we conclude that for device 1 a single 9-AGNR bridges the graphene gap (as illustrated in Figure 2a, left). In Figure S2, we present another 9-AGNR device (Device 2) with a single 9-AGNR contacted in the junction.

Figure 2b presents the same measurements as in Figure 2a, but acquired on Device 3 on which nonaligned 5-AGNRs have been transferred. The middle panel presents the stability diagram, exhibiting the characteristics of a single QD. Within the full gate range from −8 to 8 V, we observe regular and closed Coulomb diamonds with *E*_add_, between 100 and 130 meV. We note that two gate-switching events are present, as marked by black arrows. At around *V*_G1_ = 5 V, a single large diamond with the size of 260 meV is observed, which we assign to the band gap of 5-AGNR. In addition, several excited states (marked by green dashed lines) are observed, with energies of 28–42 meV. Turning to the *V*_G1_–*V*_G2_ map (Figure 2b, right), we observe multiple diagonal resonances, all with comparable slopes. A relative gate coupling of α_G1_/α_G2_ ≈ 2.1 can be extracted. We therefore conclude that, as shown in Figure 2a, a single 5-AGNR is connected between the two electrodes. In Figure S3, two additional devices (Devices 4 and 5) with single 5-AGNRs bridging the graphene gap are presented.

The second scenario that we have encountered is presented in Figure 2c,d, with transport data recorded on Devices 6 and 8. Figure 2c shows the transport data measured on Device 6 on which 9-AGNRs are contacted with the stability diagram (middle) showing several Coulomb diamonds in the measured gate range. However, there are some distinctive features compared to Devices 1–5 where a single GNR is contacted. In the gate range of (−2 to 4 V), four regular and closing diamonds with *E*_add_ of ∼60–110 meV are observed. On top of that, we observe a larger diamond (with an *E*_add_ of ∼550 meV) overlapping with the four smaller diamonds. Such an overlap of diamonds suggests that charge transport occurs via two or more QDs in parallel, each of a different size. We note that some gate-switching events occur at several gate voltages. To further investigate the geometry of QDs in the junction, we recorded the dual gate sweep (*V*_Bias_ = 1 mV), as shown in the right panel of Figure 2c. In the plot, several Coulomb resonances with two different slopes are observed. Two strong resonances are present, marked by two white arrows corresponding to the charge transitions at the left and right sides of the large diamond, respectively. In between them, four weaker resonances are identified, corresponding to the charge transitions between the four smaller diamonds with charge states *n*, *n* + 1, *n* + 2, and *n* + 3. The different slopes of the resonances confirm that more than one (in this case two) QD are formed in parallel. The two sets of mutually parallel resonances couple differently to the two gates, indicating that both QDs are located at different positions in the nanogap, as illustrated in Figure 2c, left. Moreover, no noticeable interaction is observed between the two sets of Coulomb resonances, indicating that the two QDs are independent or are only weakly coupled. In Figure S4, transport measurements for an additional device (Device 7) with 9-AGNRs are shown where multiple QDs are formed in parallel. In Figure 2d, we present evidence that also in the case of the 5-AGNRs, multiple QDs can be formed in parallel (device 8). In the stability diagram (middle panel), we observe two sets of Coulomb diamonds with different addition energies that are overlapping (one around 120 meV and another around 220 meV). Moreover, in the dual-gate current map (right panel), several resonances are observed, with predominantly two slopes.

To further analyze the QDs in the various devices, we extract the total tunnel coupling Γ of the QD to the leads by using the Breit–Wigner (BW) model for resonant transport through a single-lifetime-broadened transport level.^17^ The details of the fitting procedure are provided in Figure S6. Table 1 summarizes the key parameters obtained from Devices 1–9, including the coupling Γ, number of GNRs, band gap (*E*_gap_), addition energies excluding the one in band gap (*E*_add_), and energies of excited states (*E*_exci._). We find that the coupling Γ for the 9-AGNR-based devices is between 1.3 and 6.7 meV, while for the 5-AGNRs-based devices, it is between 6.2 and 11.5 meV. The relatively large coupling values measured in some of our devices indicate that the GNR-electrode couplings are in the intermediate regime, aligning with the appearance of the elastic cotunneling observed in Device 1. While a previous GNR/graphene device system reported significantly weaker tunnel couplings with sharp single-electron features in transport measurements,^9^ a recent work involving porphyrin nanoribbon devices exhibited an intermediate ribbon–graphene coupling of about 7 meV,^18^ similar to our devices. Moreover, we notice a large device-to-device variation, which could be due to different overlap areas between GNRs and graphene electrodes and the cleanliness of the electrodes. Interestingly, the coupling values for the 5-AGNR devices are larger than those for the 9-AGNRs. We tentatively attribute this to a cleaner transfer process for 5-AGNRs than that for 9-AGNRs (see the Supporting Information).

**Table 1:**
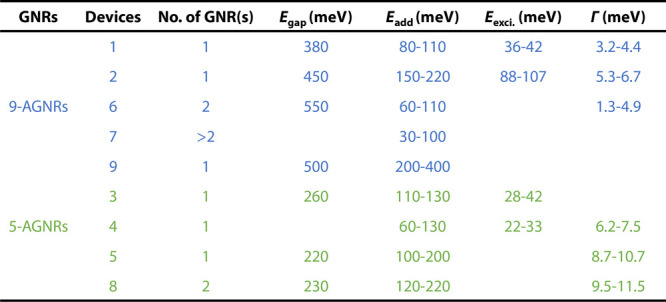
Extracted Parameters for Devices 1–9

Overall, we find that the observed band gaps for the 9-AGNRs are as high as ∼380–550 meV (Devices 1, 2, and 6) while those for 5-AGNRs are smaller, around ∼220–260 meV (Devices 3, 5, and 8). These observations follow qualitatively the same trend as predicted by quantum chemistry calculations and observed experimentally, where 9-AGNRs have a much larger band gap than 5-AGNRs (1.40 eV^19^ versus 0.1–0.2 eV,^20^ respectively). The discrepancy between our observed addition energies is attributed to different electrostatic environments,^19^ structural distortion,^21^ strain effect,^22^ or edge bond relaxation.^23,24^

In Figure 3, we present temperature-dependent transport data recorded on a 9-AGNR device (Device 9). The stability diagram recorded at 10 K (Figure 3a) shows several irregular and nonclosing diamonds with addition energies between 200 and 500 meV. Such nonclosing diamonds have been measured previously and may have different origins. Figure 3b shows the dual-gate current map measured at *V*_Bias_ = 10 mV. Several Coulomb resonances with predominantly the same slope can be seen. We therefore exclude the case of two QDs in the junction both in series and in parallel. Another mechanism that can lead to a low-bias current blockade is Franck–Condon (FC) blockade which usually possesses characteristics of equally spaced lines that run parallel to the diamond edges.^9,10,25^ Although several parallel lines are observed in the stability diagram of Figure 3a, the spacing between these lines varies significantly from 7 to 18 meV (a few selected lines are indicated by white arrows). We therefore also exclude FC blockade as a possible explanation for the nonclosing diamonds. The third possibility is the electrostatic interaction of the QD with charge traps in the gate dielectric. This effect has been studied both experimentally and theoretically. In this case, the accumulation of even a single charge in the trap can lead to a shift in the Coulomb diamond pattern while sweeping the gate, and results in a suppressed conductance in the low-bias regime.^26,27^ Here we attribute the nonclosing Coulomb diamonds in Device 9 to the QD formed in a single 9-AGNR and the interaction with a charge trap, as illustrated in Figure 3c.

**Figure 3:**
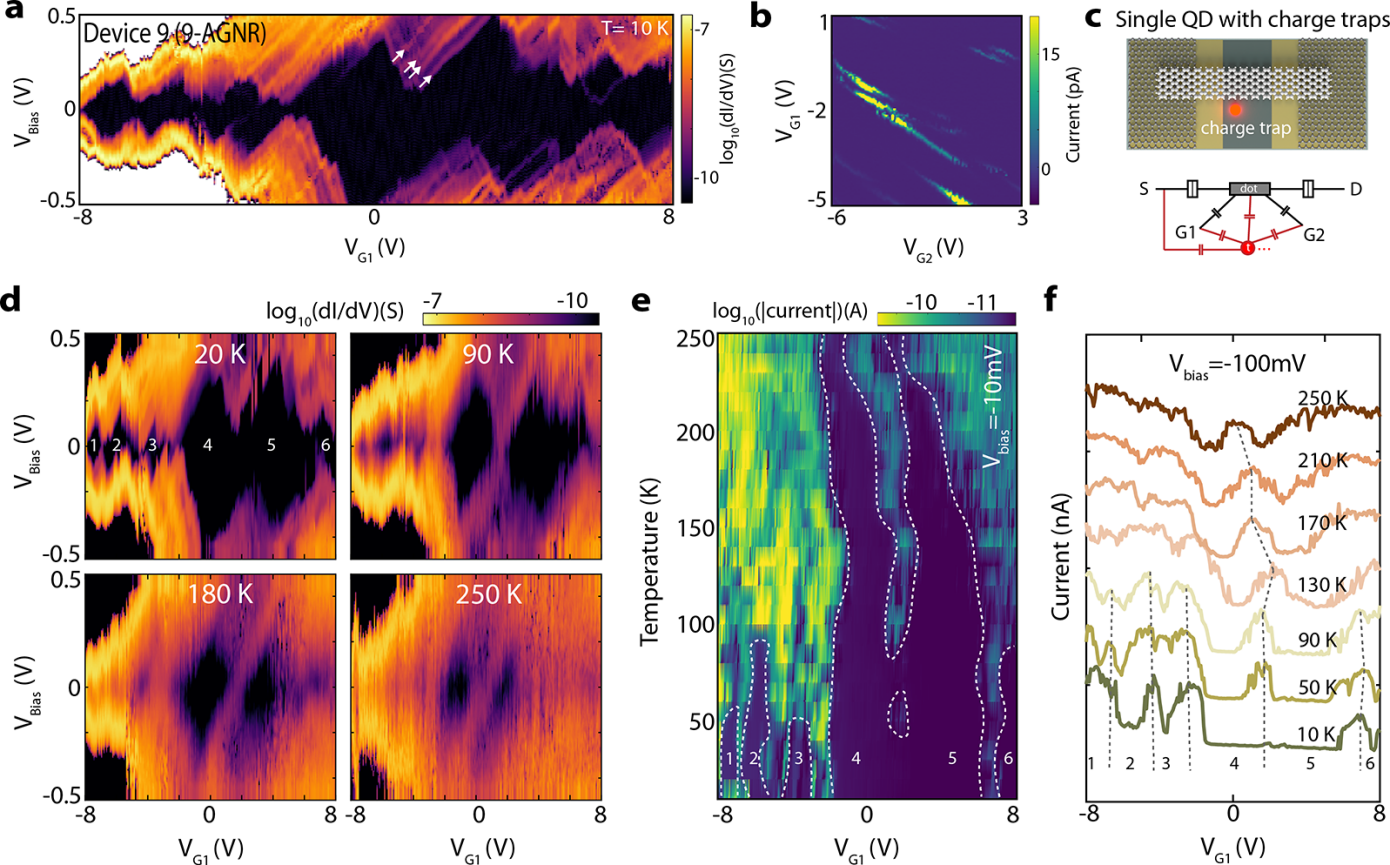
Temperature effect on QD behavior. (a–c) Coulomb blockade transport measurements on Device 9 with a geometry of a single-QD (or a single 9-AGNR) coupled with charge traps. (a) d*I*/d*V* as a function of *V*_Bias_ and *V*_G1_ on logarithmic scale recorded at *V*_G2_ = 0 V and a temperature of 10 K. A few nonequally spaced lines running parallel to the edges of the Coulomb are marked by white arrows. (b) Low-bias (*V*_Bias_ = 10 mV) current as a function of *V*_G1_ and *V*_G2_, including several Coulomb resonances in parallel. (c) Illustration of a coupled QD-charge trap geometry and the proposed equivalent circuit. (d) Selected stability diagrams measured at four different temperatures (20, 90, 180, and 250 K) recorded on Device 9. (e) Current map as a function of temperature and *V*_G1_, extracted from linecuts at *V*_Bias_ = −10 mV from temperature-dependent stability diagrams on Device 9. The edges of Coulomb blockade regions for six selected diamonds (1–6) as labeled in d are marked with white dashed lines. (f) Temperature-dependent gate sweeps along line cuts in selected temperature-dependent stability diagrams at *V*_Bias_ = −100 mV. The curves are shifted vertically to enhance visibility.

To study the effect of temperature on our GNR-based QD. We record stability diagrams at various temperatures from 10 to 250 K, with four selected stability diagrams presented in Figure 3d. At 20 K, the stability diagram shows very similar transport features as for 10 K (Figure 3a), with a few nonclosing diamonds (labeled 1–6). Upon increasing the temperature to 90 K, the small diamonds (1–3) in the negative gate regime are washed out due to thermal broadening. Another interesting change at 90K is that the two large diamonds (4 and 5) are now closed. When further increasing the temperature (180 and 250 K), the edges of diamonds 4 and 5 broaden significantly. Nevertheless, diamonds 4 and 5 are still clearly visible at temperatures up to 250 K, including the crossing point between them. The full data set (stability diagrams at all temperatures) is provided in Figure S5.

To further study the effect of temperature on the charge transport, we plot a map of the current as a function of temperature and *V*_G1_ (Figure 3e) at *V*_Bias_ = −10 mV. Similarly, Figure 3f plots several gate sweeps for a few selected temperatures at *V*_Bias_ = −100 mV. In both measurements, the edges of the Coulomb blockade regions are highlighted with dashed lines. The four small diamonds (1–3 and 6) are gradually washed out when reaching a temperature of 50–90 K. The large diamonds 4 and 5 are smeared out, but their shape persists to a temperature of 250 K. Interestingly, diamonds 4 and 5 do not close at temperatures between 10 and 40 K. However, they close at 50 K, open again when the temperature increases to 60–70 K, and eventually close for temperatures larger than 80 K. We attribute these changes in diamond shape to a population/depopulation of the charge traps with temperature, in line with observations in other QD systems.^28^ This change in the population of the charge traps could also explain the observed shifts in the positions of the Coulomb diamonds with temperature.

In conclusion, we developed a dual-gate FET architecture to contact atomically precise GNRs (9-AGNRs and 5-AGNRs), with which we can evaluate the number of GNRs contacted in the junctions and identify several scenarios that can occur in the junction: a single QD, two or multiple QDs in parallel, and a single QD close to a charge trap. Importantly, our approach enables the contacting and identification of individual quantum dot (QD) devices, a crucial aspect for harnessing the highly tunable physical properties of GNRs in devices and leveraging them for technological applications.

## Data Availability

The data sets generated during and/or analyzed during the current study are available from the authors on reasonable request.

## Supporting Information Available

Materials and methods describing device fabrication, transport data on Device 2, transport data on Devices 4 and 5, transport data on Device 7, temperature-dependent measurements, and tunnel coupling fitting (PDF)
