# Supplementary material for: Determining the Number of Graphene Nanoribbons in Dual-Gate Field-Effect Transistors
Source: Nano Lett. 2023 Sep 6;23(18):8474–80. doi: 10.1021/acs.nanolett.3c01931 (PMC10540264; doi:10.1021/acs.nanolett.3c01931)
Supplement: Supplementary file 1 — nl3c01931_si_001.pdf [file nl3c01931_si_001.pdf]

Supplementary Materials for

Determining the number of graphene nanoribbons in dual-gate field-effect transistors

Jian Zhang,[1] Gabriela Borin Barin,[2] Roman Furrer,[1] Cheng-Zhuo Du,[3] Xiao-Ye Wang,[3] Klaus Müllen,[4] Pascal Ruffieux,[2] Roman Fasel,[2,5] Michel Calame,[1,6,7] and Mickael L. Perrin[8,1,9]

[1]*Transport at Nanoscale Interfaces Laboratory, Empa, Swiss Federal Laboratories for Materials Science and Technology, 8600 Dübendorf, Switzerland*[*]

[2]*nanotech@surfaces Laboratory, Empa, Swiss Federal Laboratories for Materials Science and Technology, 8600 Dübendorf, Switzerland*

[3]*State Key Laboratory of Elemento-Organic Chemistry, College of Chemistry, Nankai University, 300071 Tianjin, China*

[4]*Max Planck Institute for Polymer Research, 55128 Mainz, Germany*

[5]*Department of Chemistry, Biochemistry and Pharmaceutical Sciences, University of Bern, 3012 Bern, Switzerland*

[6]*Department of Physics, University of Basel, 4056 Basel, Switzerland*

[7]*Swiss Nanoscience Institute, University of Basel, 4056 Basel, Switzerland*[†]

[8]*Department of Information Technology and Electrical Engineering, ETH Zurich, 8092 Zurich, Switzerland*

[9]*Quantum Center, ETH Zürich, 8093 Zürich, Switzerland*[‡]

MATERIALS AND METHODS

Graphene growth, transfer, and nanogap formation

Polycrystalline graphene is synthesized via chemical vapor deposition (CVD), transferred, and pre-patterned as reported in our previous work.[1] After a first pre-patterning of the graphene, a 60 nm thick CSAR resist (AR-P 6200.04, Allresist GmbH) is spin-coated. Following the second electron beam exposure, the resist is developed using a suitable developer (AR 600-546, Allresist GmbH) at room temperature for 1 min followed by an IPA rinse. Reactive ion etching, RIE (15 sccm Ar, 30 sccm O_2, 25 W, 18 mTorr) for 6-8 s was used to cut the graphene within the CSAR gap. After RIE, the etching mask is removed by immersing in 1-Methyl-2-pyrrolidinone (NMP) (Sigma Aldrich) at room temperature for 10 min followed by 60 min at 80 °C, cooled down for 30 min, rinsed with IPA, and blown dry with N_2. This approach yields clean and well-separated graphene electrodes (15 nm-25 nm nanogaps).

On-surface synthesis of GNRs and transfer to device substrate

9-AGNRs were synthesized from 3',6'-diiodo-1,1':2',1"-terphenyl (DITP). [2] Using a Au(788) single crystal (MaTeK, Germany) as growth substrate results in uniaxially aligned 9-AGNRs (GNRs grown along the narrow Au(111) terraces).[3] The Au(788) surface is cleaned in ultrahigh vacuum by two sputtering/annealing cycles: 1 kV Ar^+ for 10 min followed by annealing at 420 °C for 10 min. Next, the precursor monomer DITP is sublimed onto the Au(788) surface from a quartz crucible heated to 70 °C, with the substrate held at room temperature. After deposition of about 60%-70% of one monolayer DITP, the substrate is heated (0.5 K/s) to 200 °C with a 10 min holding time to activate the polymerization reaction, followed by annealing at 400 °C (0.5 K/s with a 10 min holding time) to form the GNRs via cyclodehydrogenation. The average GNR length is between 40 and 45 nm.[2] 9-AGNRs are transferred from their growth substrate to the silicon-based substrates with predefined graphene electrodes by an electrochemical delamination method using PMMA as described previously.[3–5] As the transfer of the 9-AGNRs onto the substrate exposes the graphene electrodes to water, the samples were heated to 200 °C for 120 minutes at

* jian.zhang@empa.ch
† michel.calame@empa.ch
‡ mickael.perrin@ee.ethz.ch

10^{-6} mbar to remove water residues at the graphene/GNR interface and improve the device performance.[1]

5-AGNRs were synthesized from a mixture of 3,9-diiodoperylene and 3,10-diiodoperylene (DIP) on Au (111)/mica (Phasis, Switzerland). The Au (111) surface is cleaned with similar conditions as the Au (788) single crystal described above. The precursor monomer DIP was sublimed onto the Au(111) surface from a quartz crucible heated to 200C. with the substrate held at room temperature. After deposition of one monolayer DIP, the substrate is heated (0.1 K/s) to 225 ∘C with a 5 min holding time to form the 5-AGNR. 5AGNRs are transferred from the Au(111)/mica substrate to the device substrate by a membrane-free method as described previously[6].

Electronic measurements

All electronic measurements were performed under vacuum conditions (<10^{-6} mbar). The devices were measured in two commercially available probe stations: Lake Shore Cryogenics, Model CRX-6.5 K at various temperatures (9 K - 350 K) and Lake Shore Cryogenics, Model CRX-4 K at various temperatures (4 K - 350 K). A data acquisition board (ADwin-Gold II, Jäger Computergesteuerte Messtechnik GmbH) is employed to apply the bias and gate voltages and read the voltage output of the I–V converter (DDPCA-300, FEMTO Messtechnik GmbH).

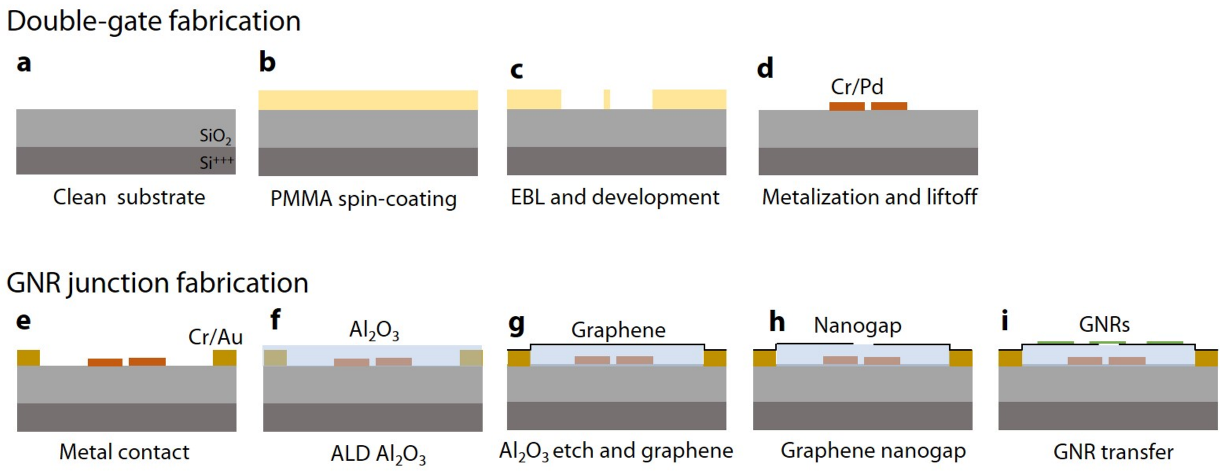

Figure S1. **Device fabrication process flow.** Top row:Double-gate fabrication. Bottom row: GNR junction fabrication. (**a**) Si/SiO_2 clean substrate. (**b**) Spin-coating of PMMA resist (yellow). (**c**) Electron beam lithography and development. (**d**) Metalization and lift-off process. (**e**) Defining and metallization of Cr/Au contacts to the graphene using EBL and subsequent lift-off process. (**f**) Deposition of 20 nm Al_2O_3 (light blue) using ALD. (**g**) Wet-etching of Al_2O_3 to open the contacts and transfer of CVD-grown graphene. (**h**) Shaping of the graphene and nanogap formation using EBL and subsequent RIE. (**i**) Transfer of GNRs.

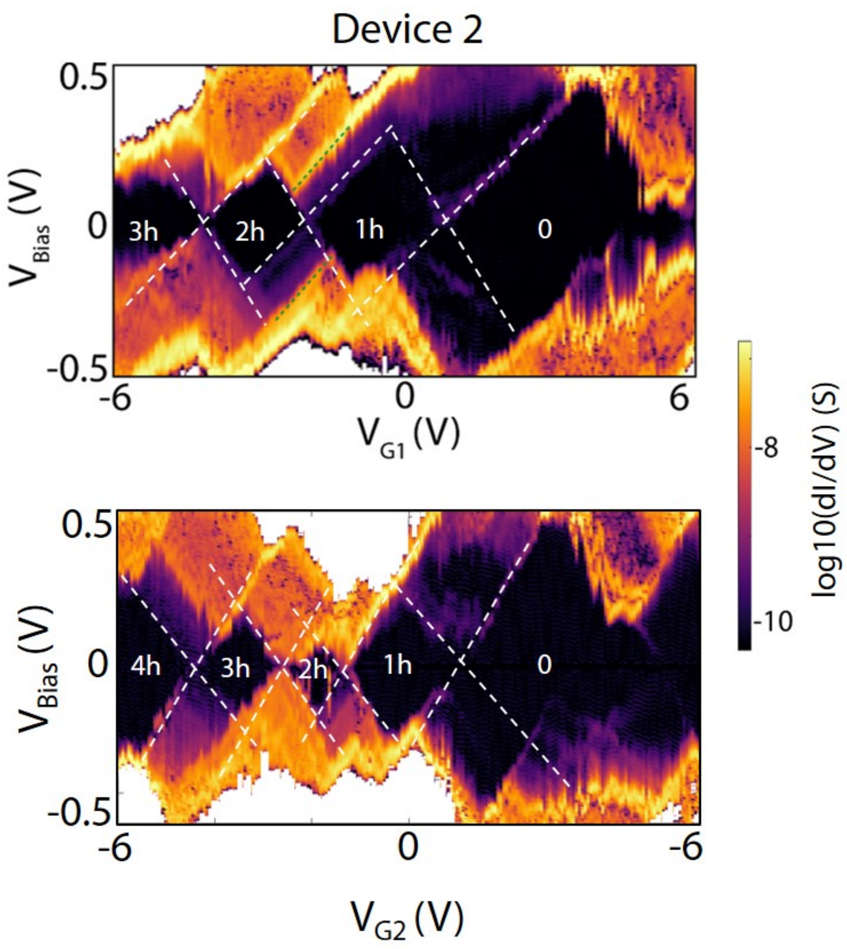

Figure S2. **Charge transport measurements on Device 2 with contacting a single 9-AGNR at 10 K.** Top: Differential conduction dI/dV as a function of V_{Bias} and V_{G1} on logarithmic scale recorded at V_{G2}=0 V.Bottom: Differential conductance (dI/dV) as a function of V_{Bias} and V_{G2} recorded at V_{G1}=0 V. The number of holes is assigned. Compared to the stability diagram measured by sweeping V_{G1}, the Stability here shows more and narrower Coulomb diamonds, indicating a stronger gate coupling of G2 to the QD than G1.

Figure S2 (top) presents the differential conductance (dI/dV) as a function of the bias voltage V_{Bias} and the gate voltage V_{G1} recorded on Device 2. Within the measured gate range, regular and closed Coulomb diamonds associated with charge transport through a single quantum dot are observed. At around V_{G1}= 2 V, a single large diamond with the size of 450 meV is observed, which we attribute to the bandgap of 9-AGNR. Based on the position of the bandgap, the number of holes in the dot is assigned. The addition energies E_{add} in

the hole regime, extracted from the diamond size, are between 150-220 meV. We observe multiple gate-switching events at the gate range of 4 V-6 V, hindering the observation of clear Coulomb diamonds at the electron regime. Moreover, two clear additional resonances with energies of 88 meV or 107 meV (green dashed line) running parallel to the diamond edge (1h-2h) are observed. These resonances correspond to an electronic or vibrational excited state of the GNR, creating an additional transport channel. The presence of regular, closing Coulomb diamonds and excited states are commonly interpreted as signatures of a single QD dominating transport, revealing that in Device 2 only a single 9-AGNR contributes to the charge transport in the available gate range. We also record the stability diagram while sweeping V_{G2} (bottom), showing qualitatively similar Coulomb diamonds, albeit with a larger gate coupling of G2 to the QD.

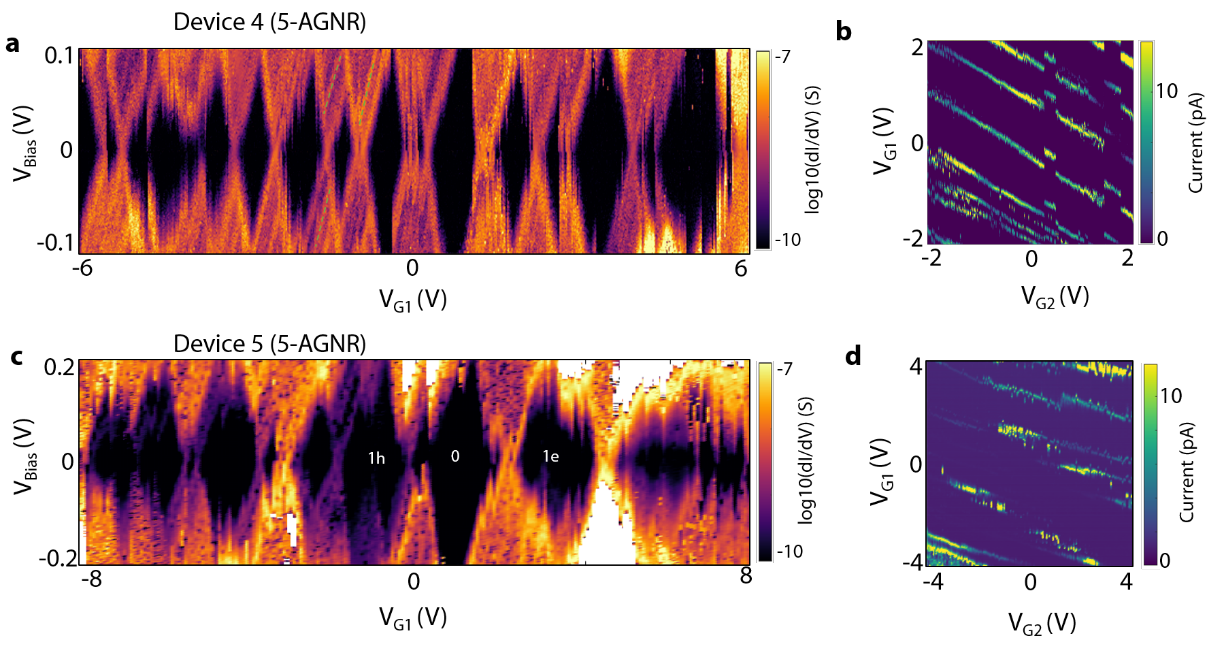

Figure S3. **Charge transport measurements on Device 4 and 5 with each a single 5-AGNR in the junction at 4 K.** (**a**) Differential conductance (dI/dV) as a function of V_{Bias} and V_{G1} recorded at V_{G2} = 0 V. In the full gate range of (-6V, 6V), several regular and closed Coulomb diamonds with E_{add} of ∼60-130 meV are observed. In addition, a few clear excited states (marked by green dash lines) are observed with energies of 22-33 meV. (**b**) Low-bias (V_{Bias} = 1 mV) current as a function of V_{G1} and V_{G2}, including several Coulomb resonances in parallel. (**c**) Differential conductance (dI/dV) as a function of V_{Bias} and V_{G1} recorded at V_{G2} = 0 V. In the full gate range of (-8V, 8V), several regular and closed Coulomb diamonds with E_{add} of ∼100-200 meV are observed. Some diamonds are distorted though. (**d**) Low bias (V_{Bias} = 1 mV) current as a function of V_{G1} and V_{G2}, including several Coulomb resonances in parallel.

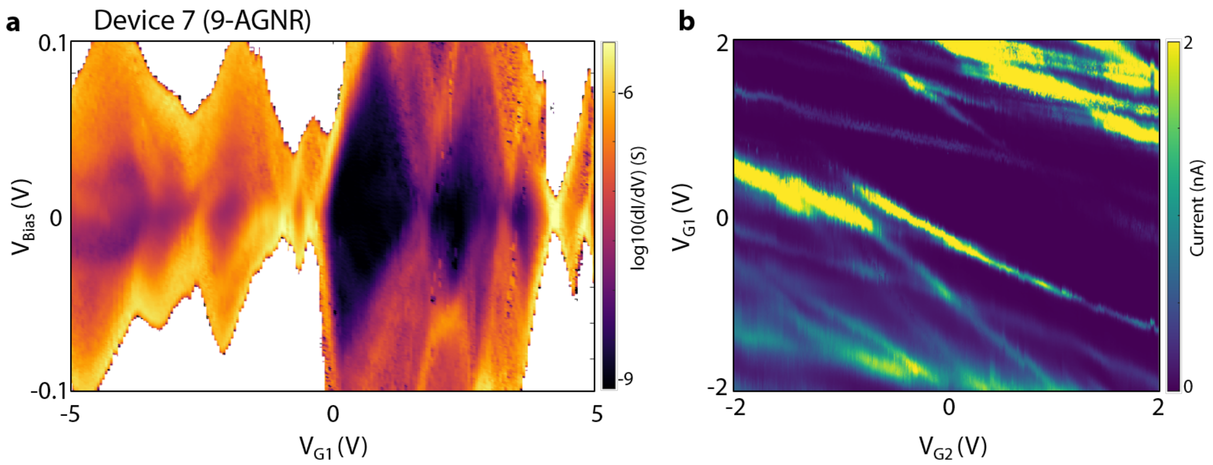

Figure S4. **Charge transport measurements on Device 7 with multiple parallel 9-AGNRs in the junction at 4 K.** (**a**) Differential conductance ($\mathrm{d}I/\mathrm{d}V$) as a function of V_{Bias} and V_{G1} recorded at $V_{\mathrm{G2}} = 0$ V. (**b**) Low-bias ($V_{\mathrm{Bias}} = 1$ mV) current as a function of V_{G1} and V_{G2}, including several Coulomb resonances in different slopes.

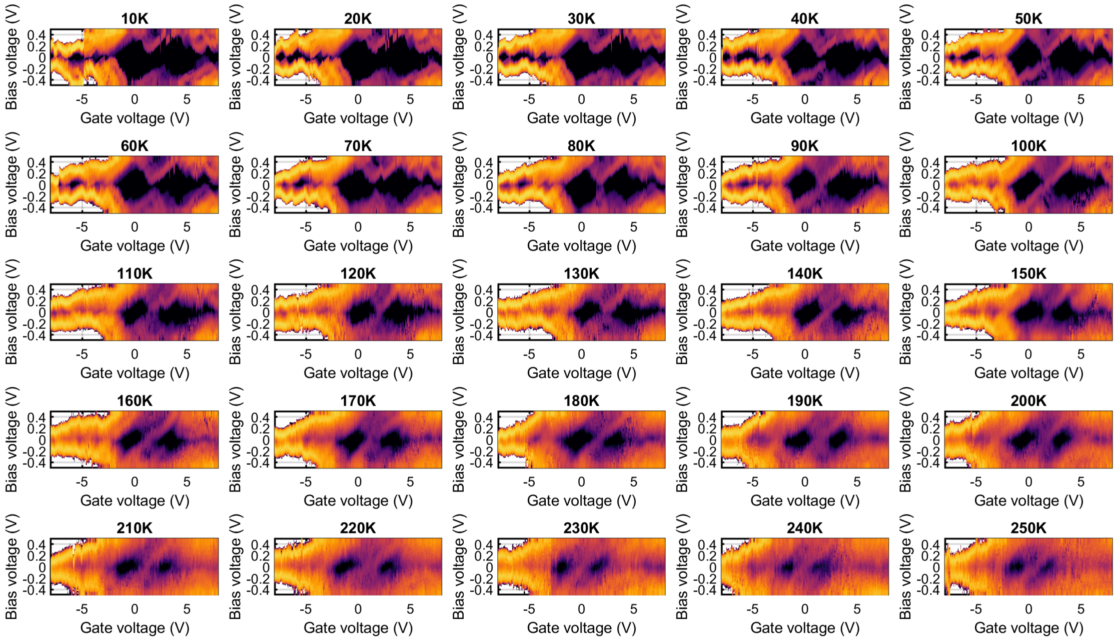

Figure S5. **Full temperature-dependent measurements of stability diagrams from 10 K to 250 K on Device 9.** Differential conductance (dI/dV) as a function of V_{Bias} and gate voltages (V_{G1}) recorded on Device 8 at various temperatures.

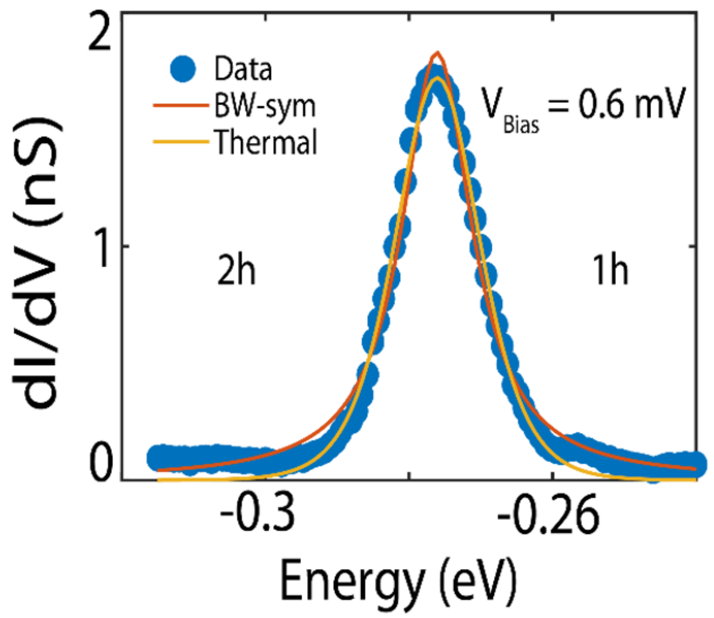

Figure S6. **An example of the tunnel coupling fitting.** Coulomb blockade resonance nonlinear fittings at a small bias ($V_{Bias} = 0.6$ mV) for the crossing point between 1h and 2h charge transition on Device 2. dI/dV data (blue points) are measured by a separated V_{G1} sweep. The solid red curve is the Breit-Wigner (BW) model fitting, the solid yellow curve is pure thermally broadened resonances fitting.

Here we show an example of the tunnel coupling fitting on the QDs in Device 2, we extract the total tunnel coupling Γ of the QD to the leads. In Fig. S6, we fit the Coulomb blockade resonance (blue dot curve) at the $1h$-$2h$ transition with the Breit-Wigner (BW) model (red curve) for resonant transport through a single-lifetime-broadened transport level.[7] From the fitting, a coupling Γ of 6 meV is extracted. To exclude that the resonances are purely temperature-broadened, we fit our data to thermally broadened resonances (yellow curve).[7] and find that the equivalent temperature is around $\sim$46 K. This is significantly higher than the cryostat temperature (10 K), indicating that the broadening of the resonances observed in our measurement is the result of the combined effect of hybridization with the electrode and temperature. The details of the fittings are provided in the following.

In the Breit-Wigner model, the peak shape is described by:[7]

$$G(\Delta V_G) = \frac{e^2}{h} \frac{\Gamma_1 \Gamma_2}{(\Delta E^2 + \frac{\Gamma^2}{4})}, \tag{1}$$

with $\Gamma = \Gamma_1 + \Gamma_2$ and the QD level detuning:

$$\Delta E = -e\alpha(\Delta V_G - V_G^{(0)}), \tag{2}$$

where $V_G^{(0)}$ is the position of the resonance. Here, α is the gate coupling of the backgate to the QD, described using the following relation:

$$\alpha = \frac{\Delta V_{Bias}}{\Delta V_G}. \tag{3}$$

The conductance of a thermally-broadened level is described as follows:[7]

$$G = \frac{e^2}{h}\frac{1}{4K_BT}\frac{\Gamma_1\Gamma_2}{\Gamma}cosh^{-2}(\frac{\Delta E}{2k_BT}), \tag{4}$$

with k_B being the Boltzmann constant and T, the bath temperature.

[1] Braun, O. *et al.* Optimized graphene electrodes for contacting graphene nanoribbons. *Carbon* **184**, 331–339 (2021).

[2] Di Giovannantonio, M. *et al.* On-surface growth dynamics of graphene nanoribbons: The role of halogen functionalization. *ACS Nano* **12**, 74–81 (2018).

[3] Overbeck, J. *et al.* Optimized substrates and measurement approaches for raman spectroscopy of graphene nanoribbons. *Physica Status Solidi (b) Basic Research* **256**, 1900343 (2019).

[4] Senkovskiy, B. *et al.* Making graphene nanoribbons photoluminescent. *Nano letters* **17**, 4029–4037 (2017).

[5] Overbeck, J. *et al.* A universal length-dependent vibrational mode in graphene nanoribbons. *ACS Nano* **13**, 13083–13091 (2019).

[6] Borin Barin, G. *et al.* Surface-synthesized graphene nanoribbons for room temperature switching devices: Substrate transfer and ex situ characterization. *ACS Applied Materials and Interfaces* **2**, 2184–2192 (2019).

[7] Gramich, J., Baumgartner, A. & Schönenberger, C. Resonant and inelastic andreev tunneling observed on a carbon nanotube quantum dot. *Physical review letters* **115**, 216801 (2015).
